# Do Conflict Resolution and Recovery Predict the Survival of Adolescents' Romantic Relationships?

**DOI:** 10.1371/journal.pone.0061871

**Published:** 2013-04-17

**Authors:** Thao Ha, Geertjan Overbeek, Anna Lichtwarck-Aschoff, Rutger C. M. E. Engels

**Affiliations:** 1 Department of Developmental Psychopathology, Behavioural Science Institute, Radboud University Nijmegen, Nijmegen, The Netherlands; 2 Department of Developmental Psychology, University of Utrecht, Utrecht, The Netherlands; Sapienza University of Rome, Italy

## Abstract

Numerous studies have shown that being able to resolve and recover from conflicts is of key importance for relationship satisfaction and stability in adults. Less is known about the importance of these relationship dynamics in adolescent romantic relationships. Therefore, this study investigated whether conflict resolution and recovery predict breakups in middle adolescent couples. Couples who are able to resolve and recover from conflict were expected to demonstrate a lower probability of breaking up. In total, 80 adolescent couples (*M* age = 15.48, *SD* = 1.16) participated in a 4-wave prospective questionnaire and observational study, with one year between measurements. In addition to self-report measures, adolescents were observed in real-time during conflicts with their partners. Multilevel Proportional Hazard analyses revealed that, contrary to the hypothesis, conflict resolution and conflict recovery did not predict the likelihood of breakup. Survival differences were not attributable to conflict resolution or conflict recovery. More research is needed to consider the unique relationship factors of adolescent romantic relationships to determine why some relationships survive while others do not.

## Introduction

Conflict and disagreements are at the heart of romantic relationships. How couples approach conflicts and especially how well partners are able to resolve conflicts affects relationship functioning and relationship stability [1]. Numerous studies have reported that couples who are unable to resolve daily conflicts have a higher likelihood of divorcing [2], [3]. Meanwhile, a study found over a period of 14 years that couples who negotiate conflicts constructively are the most satisfied and have the least chance of divorcing [4], [5]. As a result of these findings, a variety of marital therapies have been developed with a common focus on increasing couples' ability to approach and resolve conflicts constructively [6]–[9]. Given this knowledge, the relative paucity of research on conflicts in adolescents' romantic relationships is surprising [10], [11]. These early romantic relationships are thought to form a crucial social–emotional basis that underlies partner relationship quality later in life [12], [13]. In the past decade, research on adolescents' romantic relationships has increased, possibly because of the recognition that teenage romantic relationships are not trivial flings, but rather affect adolescents' mental health [14].

Research has shown that unresolved conflicts are likely to recur; if not handled well, frustration will accumulate, aggravating interaction patterns that potentially disrupt relationships. However, successfully working through issues actually promotes the relationship bond between partners [15], [16]. Previous longitudinal studies among married couples have focused on the effect of conflict resolution and conflict recovery on divorce. Conflict resolution and conflict recovery are related but distinct concepts. Conflict resolution taps into general resolution approaches during the conflict [17]; conflict recovery taps into the ability to shift out of the conflict. With regard to conflict resolution, self-report studies found that positive problem solving (i.e., constructively engaging in the conflict) is related to relationship stability whereas negative problem solving (e.g., conflict engagement, withdrawal, and compliance) is associated with lower marital quality [18], [19]. Observational research has similarly shown that high levels of negative emotions during conflict discussions predict divorce [4], [20]–[25].

More recently, researchers have focused increased attention on conflict recovery [26], [27]. Successful recovery from conflicts enables couples to refocus on new, positive relationship goals [27]. Conflict recovery is typically operationalized as the level of positive emotions after a conflict discussion (i.e., in a subsequent interaction). Gottman and Levenson [28] found that the ability to recover from conflict predicted a lower likelihood of divorce over a 4-year period [26]. Among a sample of young adults, Salvatore and colleagues [27] found that partners' ability to recover was of key importance for relationship stability over a 2-year period.

Despite the clear role of conflict resolution and conflict recovery in adult relationship maintenance, longitudinal observational and self-report studies of conflict resolution and recovery in middle adolescents' romantic relationships are lacking. This is unfortunate because the formation and maintenance of romantic relationships present important developmental tasks during adolescence [29], [30]. Early and middle adolescent years in particular may constitute a sensitive period in this respect, as teens begin to learn how to interact and handle conflicts with their romantic partners [31]. Whether conflict resolution and recovery are equally important for adolescents' relationship stability remains unclear. To our knowledge, only two previous studies have investigated conflict resolution during adolescence in relation to breakups. These have produced mixed results; one observational study [10] found that negative conflict resolution styles shorten the longevity of adolescents' romantic relationships whereas another (self-report) study found no effect of conflict resolution on breakup [32]. Conflict recovery has not been investigated in this age group.

To address this gap, we investigate whether conflict resolution and recovery predict break-up of relationships in middle adolescent couples. This is the first empirical study to use both self-report and observational methods in a longitudinal design with participants of this particular age. We hypothesized that 1) destructive conflict resolution would relate to higher probabilities of breaking up and 2) lower levels of conflict recovery would relate to higher probabilities of breaking up in middle adolescent couples.

## Methods

### Ethics Statement

The Ethical Committee of the Faculty of Social Sciences, Radboud University Nijmegen, approved the protocols and consent procedures for the present study. We obtained informed written consent from all participants involved in the study. All parents of the participants were informed about the aims of the study and were asked to provide consent for their child's participation. If they did not agree with their child's participation they could return a decline letter or contact the researcher directly (passive consent procedure). Three parents contacted the researchers for additional information, but none of them declined consent. All data was analyzed anonymously.

### Participants

A total of 1,913 adolescents between 13 and 18 years old (*M* = 15.34, *SD* = .80; *n* = 983 girls) participated in a large project examining social skills and general dating behaviors [33]. The participants were recruited from 10 secondary schools in the eastern part of the Netherlands. For this study, 701 adolescents (36.6% of the original sample) who had provided contact information and indicated a willingness to participate in a longitudinal study were approached. One criterion for inclusion was that adolescents were—at the time of inquiry—involved in a heterosexual relationship. Of the adolescents approached, 163 (23.3%) were involved in a romantic relationship, which is comparable to other Dutch samples [34]. Some adolescents who met the inclusion criterion ultimately did not participate because they ended their romantic relationship before the first study assessment. After obtaining adolescents' consent, we contacted the adolescents' partners and asked them to participate.

The final sample comprised 80 heterosexual couples with a mean age of 15.48 years (*SD* = 1.16) at Time 1. Most of the participants (96.2%) were of Dutch origin. In addition, 10.1% were involved in lower vocational education, 32.3% in intermediate general education, 53.8% in the highest level of secondary school (i.e., preparatory college and university education), and 3.8% in other education. We performed independent *t*-tests to examine whether sample characteristics differed between the total sample and this observation sample. No significant differences emerged regarding age, gender, origin, and level of education. Mean duration of the current relationship at Time 1 was 7.83 months (*SD* = 6.13); 56.0% of the participants had been in a relationship for less than 6 months. Regarding relationship experience at Time 1, 85.0% had been in at least one previous romantic relationship, and both girls and boys reported an average of more than 3 previous relationships (*M* = 3.8, *SD* = 2.17 and *M* = 3.3, *SD* = 1.65, respectively). We had a high retention rate, with 79 (98.8%) and 78 (97.5%) couples participating during the second and third waves, respectively. Between Time 1 and Time 2, 43 couples (53.8%) dissolved their relationships; in addition, 54 couples (67.5%) ended their relationships between Time 1 and Time 3 and 68 couples (85.0%) broke up between Time 1 and Time 4. Adolescents were paid €15 each for completing the questionnaire and participating in the observational component at every measurement.

### Procedure

One week before the observation sessions, both partners completed the questionnaire online. In the instructions, we emphasized that answers would not be given to any third party, including parents, teachers, or partners. We instructed adolescents to fill out the questionnaire individually at home and not to consult others.

Adolescents and their partners were also observed and videotaped in a private room at one of the participant's schools. Prior to the series of interactions, both adolescents were asked to independently choose the most applicable conflict subject from a list of eight common conflict issues occurring between adolescent romantic partners [35]. These conflict topics included not being on time/forgetting appointments, experiencing jealousy, parents not liking the partner, disliking friends, cheating with or kissing someone else, having to follow parental rules about dating, taking partners to parties, and dealing with money issues. The partners subsequently participated in 5 interaction tasks lasting 4.5 minutes each. After 4 minutes, the couple would hear a knock on the door (i.e., a perturbation), which served as a signal for them to resolve the conflict within the remaining 30 seconds.

Each topic was introduced separately by the researcher, who then left the room. As a warm-up task, the couple discussed a hypothetical situation in which they had won one million euros in the lottery and could spend this money. In a second, neutral task, they planned a party together. In the third discussion, the boy's conflict topic was discussed; in the fourth discussion, the girl's conflict topic was discussed. Finally, in a fifth, positive task, the adolescents discussed past shared happy memories or fun times in the relationship [36], [37].

The couples were contacted four times, with intermittent one-year time intervals. At every measurement, adolescents participated with the same partner as at Time 1 or, in the case of a new relationship, their new partner, who would be included in the study. Because we wanted to investigate how conflict resolution and recovery impacted the likelihood of relationship dissolution, we followed each of the 80 original couples at Time 1 until their break up, not including new relationships formed after Time 1. At Times 1, 2, and 3, couples completed the questionnaires online and participated in the observational study. At Time 4, we assessed adolescents' relationship status.

### Coding Procedures

The video recordings were coded using Observer software (The Observer, version 5) and a simplified 10-code version of the Specific Affect Coding (SPAFF) [38] instead of the original 18 codes [39]. Behaviors were coded in real time for each adolescent separately. This means that coders continuously defined expressed behaviors using an emotion code. Each emotion code was based on a combination of facial expressions, gestures, and speech characteristics, such as tone, volume, and speech rate. The modified SPAFF system consisted of 10 mutually exclusive emotion codes: contempt, anger, fear/anxiety, sadness/withdrawal, whining/complaining, neutral state, interest/curiosity, humor, joy/excitement, and affection. Using this system, trained observers entered codes for both adolescents independently in real time, yielding two synchronized streams of continuous data.

Before initiating coding of the video interactions, observers were intensively trained by the first author for 4 months until they reached a minimum of 75.0% agreement and .65 kappa using a frequency/sequence-based comparison and 80.0% agreement using a duration/sequence-based comparison (Noldus Observer 5.0). These two reliability methods were used to ensure accuracy in coding both at the onset and throughout the duration of the events. Weekly recalibration training was conducted to minimize coder drift. Thirty percent of all sessions were coded by two or three coders. Coders were blind to which interactions were used to assess observer agreement. In addition, the first author randomly checked the SPAFF codes of three remaining interactions every week. The average coder agreement was 81.0% (κ = .77) and 94.0% duration/sequence based.

### Measures

#### Self-reported conflict resolution

We administered the Conflict Resolution Style Inventory (CRSI; Kurdek, 1994) [40] to measure adolescents' style of handling conflicts in the current romantic relationship. The CRSI distinguishes four conflict resolution styles: conflict engagement (e.g., launching personal attacks), positive problem solving (e.g., finding alternatives that are acceptable to both partners), withdrawal (e.g., remaining silent for long periods of time), and compliance (e.g., not being willing to stick up for oneself). The CRSI has demonstrated good reliability and validity, and it has been shown that it meaningfully assessed the four conflict resolution styles in a Dutch sample of adolescents [41]. Each category was assessed using five items answered on a 5-point scale, ranging from 1 (never) to 5 (always). A mean score was calculated based on conflict engagement, withdrawal, and compliance to tap into negative conflict resolution styles [32]. Cronbach's alphas for boys and girls were high at all three waves for positive problem solving (ranging between .79 and .93) as well as negative conflict resolution styles (ranging between .73 and .89).

#### Self-reported satisfaction with actual conflict resolution

Directly after the observation, adolescents rated on a 5-point scale the extent to which they agreed about a solution with their partners. This was done separately for the two conflict discussions. Responses ranged from 1 (totally agreed) to 5 (not agreed at all). In addition, adolescents rated to what extent they felt the problem during the discussion has been resolved. Again, this was done for the two conflict discussions separately on a 5-point scale ranging from 1 (absolutely resolved) to 5 (absolutely not resolved). A mean score of these four self-ratings was used. Cronbach's alphas for boys and girls were moderate to high at all three waves (ranging between .63 and .84).

#### Expressed negativity during conflict

Adolescents' negative emotions during the boys' and girls' conflict discussions (the third and fourth discussion tasks, respectively) were used to measure the impact of the conflict discussions. Negative emotions consisted of contempt, anger, fear/anxiety, sadness/withdrawal, and whining/complaining. Total duration was calculated for negative emotions separately for boys and girls. To increase the reliability of the negative emotions scores, we aggregated both conflict discussions into a single score. Observation time was increased to improve the estimate of the interpersonal characteristic [42].

#### Conflict recovery

Positive emotions were used to measure the degree to which adolescents were able to recover from the conflict discussions. Positive emotions consisted of interest/curiosity, humor, joy/excitement, and affection. We used three indicators to tap into conflict recovery, which we calculated for boys and girls separately. First, we calculated the total duration of positive emotions after the knock on the door, which was the sign to resolve the conflict in the final 30 seconds of the conflict discussion. This measure tapped into an immediate recovery after a perturbation. Again, positive emotions were aggregated for both conflict discussions into a single score. Second, we calculated the total duration of positive emotions during the positive discussion that followed the conflict discussion to capture the extent to which the couple was able to focus on the positive discussion. Third, we calculated the difference in scores between positive emotions during the positive task and the conflict discussions. More specifically, the aggregated total duration of positive emotions of both conflict discussions was subtracted from total duration of positive emotions during the positive task. Higher values indicated that adolescents displayed more positive emotions during the positive task relative to their level of positive emotions during the conflict task.

#### Relationship status

Relationship status was assessed at Times, 2, 3, and 4 by asking both members of the couple whether they were still together with the same partner. No differences between boys and girls were recorded.

### Strategy of Analysis

To test whether self-reported and observed conflict resolution and observed conflict recovery predicted the end of middle adolescents' relationships, a Survival Analysis framework was applied [43] using the software package MPLUS 5.1 [44]. Within a Multilevel Proportional Hazard Model (Cox regression), a couple's breakup represented an event while the different measures for conflict resolution and conflict recovery across the measurement waves were specified as time-varying predictors. Thus, observed and self-reported conflict resolution as well as the observed conflict recovery was used to predict breakups in subsequent measurement waves. Separate models were estimated to examine the effects of the different time-varying predictors on the likelihood of breakups. Due to multiple tests, a Bonferroni correction was applied with α = .001. Boys' and girls' measures were always entered separately. Interaction terms between boys' and girls' measures were also tested. However, none of these were significant. Therefore, only the results of the models with main effects are reported. Because participants' age and the duration of the relationship could be related to the breakup, we controlled for these variables in all our analyses. Thus, boys' and girls' age at Time 1 and duration of the relationship at Time 1 were included as time-fixed predictors. Hazard ratios and confidence intervals were reported as effect sizes.

## Results

### Manipulation Check

To test whether we successfully elicited conflict in the paradigm employed, we conducted a repeated measures ANOVA with negative emotions in the four discussion tasks (planning a party, conflict boy, conflict girl, happy memory discussion) as a within-subject factor. A repeated measures ANOVA with a Greenhouse-Geisser correction showed that mean negative emotions differed significantly among the four discussion tasks: *F*(2.54, 404.15) = 18.78, η_p_^2^ = .11, *p*<.001. Post-hoc tests using the Bonferroni correction revealed that both conflict discussions elicited more negative emotions. Boys' conflict discussion elicited more negative emotions compared to the “planning a party” discussion (resp., *M* = 8.07, *SD* = 9.25, *M* = 3.96, *SD* = 4.83, *p*<.001) and the happy memory discussion (*M* = 4.78, *SD* = 6.20, *p*<.001). Similarly, girls' conflict discussion elicited higher levels of negative emotions than the “planning a party” discussion (*M* = 8.13, *SD* = 10.08, *p*<.001) and the happy memory discussion (*p*<.001). Levels of negative emotions were not significantly different in the boys' and girls' conflict discussions (*p* = .94) nor between the “planning a party” discussion and “happy memory” discussion (*p* = .09). Thus, conflict was successfully elicited in the conflict discussions.

### Descriptives

Independent *t*-tests showed that girls expressed significantly more negative emotions during the conflict discussions at Time 1 and Time 3 than boys (Table 1). Boys and girls did not differ on the remaining measures.

**Table 1:** Means and Standard Deviations.

	Boys		Girls			
	*M*	*SD*	*M*	*SD*	*t*	*p*
*Conflict resolution*						
Positive problem solving T1	3.78	.71	3.65	.76	1.09	.28
Positive problem solving T2	3.70	.82	3.78	.82	−.38	.71
Positive problem solving T3	3.77	.86	3.69	.85	.30	.76
Negative resolution style T1	1.75	.48	1.76	.44	−.07	.95
Negative resolution style T2	2.10	.55	1.96	.41	1.23	.22
Negative resolution style T3	1.93	.43	1.98	.39	−.40	.69
Satisfaction with actual resolution T1	2.16	.77	2.04	.79	1.05	.30
Satisfaction with actual resolution T2	2.50	.86	2.41	.83	.45	.65
Satisfaction with actual resolution T3	2.45	.70	2.25	.96	.81	.42
Expressed negativity T1	6.31	8.13	9.89	8.50	−2.73	.007
Expressed negativity T2	4.20	5.08	5.97	6.06	−1.35	.18
Expressed negativity T3	4.93	3.64	10.42	8.16	−3.01	.004
*Conflict Recovery*						
Positive emotions after perturbation T1	3.20	3.88	2.43	2.23	1.54	.13
Positive emotions after perturbation T2	2.43	2.50	2.21	2.76	.36	.72
Positive emotions after perturbation T3	2.45	2.17	2.25	2.65	.28	.78
Positive emotions after conflict T1	21.44	16.44	22.56	16.15	−.44	.66
Positive emotions after conflict T2	19.30	15.17	19.38	11.06	−.03	.98
Positive emotions after conflict T3	21.25	14.69	20.75	13.48	.12	.90
Difference score positive emotions T1	−3.50	14.76	−.87	18.15	−1.00	.32
Difference score positive emotions T2	−3.12	16.28	1.06	14.72	−1.15	.26
Difference score positive emotions T3	−3.09	10.78	.63	15.16	−.98	.33

*Note*. Duration values for observation predictors are in seconds.

### Does Conflict Resolution Predict Breakups?

Table 2 displays the results of the Multilevel Proportional Hazard Model analyses regarding conflict resolution and conflict recovery.

**Table 2:** Results for the Multilevel Proportional Hazard Model analyses on breakup regarding conflict resolution and conflict recovery.

	*b*	*SE*	*HR*	*95% CI*	*p*
*Conflict resolution*					
Boys' positive problem solving	−.27	.24	.76	.48–1.21	.26
Girls' positive problem solving	.15	.25	1.16	.72–1.87	.55
Boys' age	−.01	.10	.99	.81–1.21	.92
Girls' age	−.38	.16	.68	.50–.94	.02
Relationship duration	−.02	.02	.99	.94–1.03	.50
Boys' negative resolution style	−.11	.23	.89	.57–1.40	.63
Girls' negative resolution style	−.12	.24	.89	.55–1.43	.63
Boys' age	−.05	.04	.95	.89–1.02	.17
Girls' age	−.37	.17	.69	.50–.96	.03
Relationship duration	−.01	.02	.99	.95–1.03	.61
Boys' satisfaction with actual resolution	.07	.23	1.07	.68–1.68	.76
Girls satisfaction with actual resolution	−.05	.27	.61	.36–1.05	.08
Boys' age	−.05	.03	.96	.90–1.01	.11
Girls' age	−.30	.15	.74	.56–.98	.04
Relationship duration	−.01	.02	.99	.96–1.03	.80
Boys' expressed negativity	.19	.23	1.21	.77–1.90	.40
Girls' expressed negativity	−.42	.24	.66	.41–1.04	.07
Boys' age	−.04	.03	.96	.91–1.02	.23
Girls' age	−.34	.15	.72	.54–.95	.02
Relationship duration	−.01	.02	.99	.95–1.03	.58
*Conflict recovery*					
Boys' positive emotions after perturbation	−.65	.23	.53	.33–.82	.001
Girls' positive emotions after perturbation	−.01	.20	.99	.67–1.48	.97
Boys' age	−.07	.03	.93	.87–.99	.03
Girls' age	−.32	.15	.73	.54–.97	.03
Relationship duration	−.01	.02	.99	.96–1.03	.67
Boys' positive emotions after conflict	−.34	.21	.71	.47–1.08	.12
Girls' positive emotions after conflict	−.15	.26	.86	.52–1.43	.57
Boys' age	−.04	.03	.96	.91–1.02	.18
Girls' age	−.31	.15	.74	.55–.98	.04
Relationship duration	−.01	.02	.99	.95–1.03	.52
Boys' difference score positive emotions	.34	.22	1.41	.93–2.14	.11
Girls' difference score positive emotions	−.19	.21	.83	.55–1.25	.37
Boys' age	−.05	.03	.95	.89–1.01	.09
Girls' age	−.35	.15	.71	.52–.96	.02
Relationship duration	−.01	.02	.99	.96–1.03	.73

#### Self-reported conflict resolution

Self-reported positive problem solving was not related to a lower likelihood of breakups; neither boys' self-reported positive problem solving (Hazard Ratio = 0.76, *p* = .26, 95% CI = .48–1.21) nor girls' self-reported positive problem solving (Hazard Ratio = 1.16, *p* = .55, 95% CI = .72–1.87) predicted breakups. None of the control variables (i.e., boys' and girls' age and the duration of the relationship) were significant. In addition, neither boys' negative conflict resolution styles (Hazard Ratio = .89, *p* = .63, 95% CI = .57–1.40) nor girls' negative conflict resolution styles (Hazard Ratio = .89, *p* = .63, 95% CI = .55–1.43) predicted breakups. None of the control variables were significant.

#### Self-reported satisfaction with actual conflict resolution

Satisfaction with the actual conflict resolution was not related to a lower likelihood of breakups; neither boys' self-reported satisfaction (Hazard Ratio = 1.07, *p* = .76, 95% CI = .68–1.68) nor girls' self-reported satisfaction (Hazard Ratio = .61, *p* = .08, 95% CI = .36–1.05) predicted breakups. None of the control variables were significant.

#### Observed expressed negativity during conflict

Expressed negative emotions were not related to breakups; neither boys' negative emotions (Hazard Ratio = 1.21, *p* = .40, 95% CI = .77–1.90) nor girls' negative emotions (Hazard Ratio = .66, *p* = .07, 95% CI = .41–1.04) predicted breakups. None of the control variables were significant.

### Does Conflict Recovery Predict Breakups?

Next, we investigated whether the likelihood of breaking up depended on conflict recovery. Results indicated that boys' ability to immediately recover after a perturbation was not related to a breakup (Hazard Ratio = .53, *p* = .006; 95% CI = .33–.82), which was also true for girls (Hazard Ratio = .99, *p* = .97, 95% CI = .67–1.48). None of the control variables were significant. In addition, positive emotions after the conflict discussion were not significantly related to breakups for either boys (Hazard Ratio = .71, *p* = .12, 95% CI = .47–1.08) or girls (Hazard Ratio = .86, *p* = .57, 95% CI = .52–1.43). None of the control variables were significant. Finally, the difference score between positive emotions during the positive task and the conflict discussion was not related to breakups for either boys (Hazard Ratio = 1.41, *p* = .11, 95% CI = .93–2.14) or girls (Hazard Ratio = .83, *P* = .37, 95% CI = .55–1.25). None of the control variables were significant.

## Discussion

The results from this prospective study of adolescent couples suggest that conflict resolution and conflict recovery are not related to adolescents' romantic relationship breakups. Adolescents who were capable of either resolving or recovering from conflict were not more likely to stay together over time. These results sharply contrast the outcomes of many previous findings among late adolescents, young adults, and married couples, which provided strong evidence for the importance of resolution and recovery for relationship longevity [4], [5], [27].

Methodologically, we rigorously operationalized conflict resolution and conflict recovery, using both adolescents' self-reports and observational data. We further extended previous research by employing a longitudinal design in which stable adolescent couples were measured every year, decreasing the time between measurement and breakup. Previous studies have often relied upon a single measurement, with as many as 14 years occurring between measurement waves in marital research [4]. However, between the ages of 15 and 18, teenagers are developing quickly in their romantic relationships. As a result, features such as conflict resolution and recovery might change dramatically during this developmental period [45]. Thus, the current approach resulted in more reliable estimators of conflict resolution and recovery than when only a single measurement of conflict resolution and recovery was used to predict breakups.

Given that the current study was the first to use both self-reports and observational indicators with this particular age group, our results suggest that during this specific developmental period conflict resolution and recovery are not predictive of breakups. Previous studies have found that conflicts do occur in this age group [46], [47], and conflicts with partners tend to increase during this period as adolescents become closer and more intimate with one another [48]. As such, how can we explain that adolescents' conflict resolution and recovery are not predictive of breakup? The context in which these conflicts occur is quite different from more established relationships in early adulthood and marital relationships. Within established couples, overcoming conflict enables the couples to attain long-term relationship goals that will be reflected in higher levels of relationship satisfaction [15] and, thus, longer relationships.

However, for adolescents, the relationship context differs in two important ways. First, although conflicts increase during this developmental period for adolescents and their partners, it is unlikely that adolescents have as many conflicts as adults in marital relationships. When irritations or conflicts do occur, adolescents tend to deny or downplay the significance of them [10], [49], [50] thereby minimizing the effect of conflict on the relationship. Consequently, tapping into conflict resolution and recovery when conflicts do not occur on a regular basis might not reflect real-life significance for adolescents' romantic relationships and hence, is not predictive of dissolution.

Second, how conflicts are handled may not define adolescents' sense of relationship satisfaction. In many romantic relationships among adolescents, goals are less about long-term commitments and attachment [51]. Rather, affiliative functions of dating are more valued, such as spending time together, providing companionship, and seeking peer approval [52]–[54] as well as experiencing intimacy, passionate love, and attraction [55]. Whereas in adulthood satisfaction is experienced when partners have a daily life that reflects the long-term goals of the couple, adolescents' satisfaction might be more about recreational purposes and experiencing passionate love and attraction in the first place. Hence, during this developmental period, conflict resolution and recovery are not important yet. Over time, as the relationship goals change to support long-term commitments, conflict resolution and recovery might be more significant in defining relationship satisfaction and would therefore relate to breakups.

Several limitations of this study must be noted. First, although the current sample size is comparable to other observational studies [27], it did not allow for the investigation of potential important moderators such as frequency and severity of conflict. Second, most adolescents were of Dutch origin and engaged in intermediate or higher educational levels, which limits the generalizability of the current findings. Third, the analytical approach enabled us to make more reliable estimates of conflict resolution and recovery over a 4-year period. However, we currently know little about when conflict occurs between partners, and it is possible that conflicts within adolescents' romantic relationships might occur right before the end of the relationship. A longitudinal design in which couples are assessed monthly to predict breakups over a one-year period could shed more light on conflict resolution and conflict recovery as predictors for breakups.

Despite these limitations, this is the first prospective study to investigate conflict resolution and recovery in relation to middle adolescents' romantic breakups. These results indicate that teenagers' romantic relationships are a unique phase in development in which relationship processes that have been found to be of key importance in later periods of life are not valid. Romantic dissolutions can be a huge personal loss for teenagers, fueling feelings of despair and depressive mood. Thus, understanding how relationship factors contribute to these events needs to be better understood.
